# Not all cognitive offloading is equal: distinguishing dependent and autonomous offloading to generative AI

**DOI:** 10.3389/fpsyg.2026.1878629

**Published:** 2026-07-16

**Authors:** Qiuhan Zhu, Xiangnan Li, Yiang Dong, Pengcheng Chang, Mengmeng Fan

**Affiliations:** 1 School of Kinesiology and Physical Education, Zhengzhou University, Zhengzhou, China; 2 School of Management, Zhengzhou University, Zhengzhou, China

**Keywords:** autonomous offloading, cognitive agency transfer, cognitive offloading, dependent offloading, generative AI, intrinsic motivation

## Abstract

Generative AI tools such as ChatGPT now perform core cognitive operations—reasoning, synthesis, evaluation, and creative generation—on users' behalf, raising urgent questions for educational psychology about how AI use relates to cognitive development. Yet research on cognitive offloading has largely treated AI use as a unidimensional phenomenon, obscuring a theoretically consequential distinction: whether AI substitutes for the user's own thinking or scaffolds it. Drawing on the autonomous–dependent help typology and self-determination theory, the present study introduces and examines a distinction between *dependent cognitive offloading* (delegating core thinking to AI) and *autonomous cognitive offloading* (using AI as a scaffold while retaining cognitive agency). In a three-wave time-lagged survey study (*N* = 589 university students and early-career knowledge workers), we tested a dual-pathway model linking the two offloading modes to four perceived downstream cognitive outcomes—autonomous capability, creativity, deep processing, and independent judgment—through cognitive agency transfer and intrinsic motivation. Dependent offloading was positively associated with cognitive agency transfer and negatively associated with intrinsic motivation, which in turn were linked to poorer perceived outcomes. Autonomous offloading was positively associated with intrinsic motivation and more favorable perceived outcomes. Metacognitive monitoring attenuated the link between dependent offloading and cognitive agency transfer but did not buffer the negative motivational association. Notably, both offloading modes yielded comparable immediate benefits despite divergent downstream correlates, suggesting that potentially maladaptive AI engagement may be difficult for users to detect from immediate experience. These findings highlight the *manner* of AI engagement—not merely its frequency—as a key factor in understanding its associations with perceived cognitive functioning, and point to practical strategies for educators, learners, and AI tool designers seeking to harness AI without undermining cognitive autonomy.

## 1 Introduction

Generative artificial intelligence (AI) tools—such as ChatGPT, Claude, and Gemini—do not merely store or retrieve information; they produce coherent text, generate solutions, and construct arguments, effectively performing core cognitive operations on behalf of the user (Doshi and Hauser, 2024; Noy and Zhang, 2023). This capability means that the question is no longer simply *whether* individuals offload cognitive work to external tools (Risko and Gilbert, 2016), but whether AI is *substituting* human thinking or *scaffolding* it—and whether these two modes of engagement are associated with different consequences.

This shift surfaces a central paradox that motivates the present study. In the moment, delegating cognitive work to generative AI is almost unambiguously beneficial: tasks are completed faster, with less effort, and often to a higher apparent standard (Noy and Zhang, 2023; Doshi and Hauser, 2024). Yet the very operations now being delegated—reasoning, synthesis, evaluation, and creative generation—are the higher-order processes through which learning and professional expertise are built (Firth et al., 2019,?). The immediate utility of offloading and its potential long-term cognitive cost thus pull in opposite directions, and—because the reward is immediate while the cost, if any, is deferred and diffuse—users have little experiential basis for telling adaptive from maladaptive engagement. We argue that what determines which side of this paradox a given pattern of use falls on is not *how much* a person offloads but *how* they offload (Carter, 2018). Existing research, however, has predominantly treated cognitive offloading as a unidimensional construct (Risko and Gilbert, 2016), obscuring a consequential distinction: individuals who let AI substitute their thinking likely engage in a different cognitive process than those who use AI as a scaffold while retaining cognitive agency.

To address this gap, we introduce a distinction between *dependent cognitive offloading* and *autonomous cognitive offloading*. Dependent offloading occurs when individuals delegate core thinking to AI—accepting AI-generated answers with minimal evaluation, allowing AI to structure their reasoning, and relying on AI outputs as final products. Autonomous offloading, by contrast, occurs when individuals use AI as a scaffold—leveraging AI outputs to stimulate their own ideas, comparing AI-generated content with their own reasoning, and integrating AI assistance into self-directed cognitive activity. This distinction parallels the autonomous–dependent help typology (Nadler, 1997, 2015), which shows that the *manner* in which help is delivered—not merely its content—shapes whether recipients benefit or suffer in subsequent tasks (Nadler, 2015; Li et al., 2026).

We propose that these two offloading modes engage different psychological mechanisms. Drawing on self-determination theory (Deci and Ryan, 1985; Ryan and Deci, 2017), we argue that dependent offloading may undermine the basic psychological needs for autonomy and competence, thereby diminishing *intrinsic motivation*. Simultaneously, by habitually ceding judgment authority to AI, dependent offloading may promote *cognitive agency transfer*—a process in which individuals progressively hand over not merely the *execution* of cognitive tasks but the *governance* of cognition itself: decisions about what information matters, which perspectives to adopt, and what conclusions to draw. These mechanisms, in turn, may be linked to poorer perceived downstream cognitive outcomes. Autonomous offloading, conversely, appears to preserve intrinsic motivation by supporting autonomy and competence, without producing cognitive agency transfer.

If the paradox above is sustained by an immediate reward that masks deferred costs, then the individual capacity to see through that reward becomes pivotal. We therefore examine *metacognitive monitoring*—the capacity to evaluate one's own understanding, detect overreliance on external tools, and regulate cognitive engagement (Flavell, 1979; Grinschgl et al., 2021)—as a psychological shield against the immediate-reward trap, and as a boundary condition that may attenuate the unfavorable correlates of dependent offloading. As we develop below, this protective role is likely to be pathway-specific rather than uniform.

The present study addresses this gap through a three-wave time-lagged survey (*N* = 589) that empirically examines a mode-based account of cognitive offloading to generative AI. Our contributions are primarily empirical. First, we provide evidence that dependent and autonomous cognitive offloading are distinct dimensions of AI engagement with divergent downstream correlates—the manner of AI use, not merely its frequency, matters. Second, we identify cognitive agency transfer and intrinsic motivation as parallel mechanisms linking different offloading modes to perceived cognitive outcomes, integrating self-determination theory and the helping literature into the human–AI interaction domain. Third, we show that both offloading modes yield comparable immediate benefits despite divergent downstream correlates, suggesting that potentially maladaptive AI engagement may be difficult to detect from the user's immediate experience. We position this work as an exploratory empirical study that provides initial correlational evidence, rather than definitive construct validation or causal claims.

## 2 Theoretical background and hypotheses

### 2.1 Cognitive offloading in the era of generative AI

Cognitive offloading refers to the use of external resources to reduce internal cognitive demands (Risko and Gilbert, 2016). This phenomenon encompasses a broad range of behaviors, from writing reminders to using calculators, and is understood as a fundamental aspect of human cognition—humans routinely extend their cognitive capabilities through environmental scaffolding (Risko and Gilbert, 2016; Barr et al., 2015). Research on cognitive offloading has demonstrated that reliance on external tools can alter internal cognitive processes: individuals who outsource memory to AI show reduced encoding and inflated confidence (Sparrow et al., 2011; Fisher et al., 2015), and metacognitive beliefs about one's own ability shape the decision to offload (Boldt and Gilbert, 2019; Grinschgl et al., 2021). Importantly, has argued that offloading does not uniformly undermine memory, suggesting the need for a more differentiated view of when offloading helps vs. harms.

The advent of generative AI substantially expands the scope of cognitive offloading. While search engines offload *memory* and calculators offload *computation*, generative AI can offload *reasoning, synthesis, evaluation, and creative generation* (Noy and Zhang, 2023; Doshi and Hauser, 2024). Users can instruct AI to draft essays, construct arguments, evaluate alternatives, and propose solutions—activities that constitute the core of higher-order cognition (Woodruff et al., 2024). The scale of this shift is reflected in growing concerns about AI-induced deskilling (Shukla et al., 2025), cognitive and behavioral dependency on generative AI (Gerlich, 2025; Yu et al., 2024), and intellectual erosion (Jose et al., 2025). This qualitative shift calls for a more differentiated understanding of how distinct patterns of offloading relate to cognitive functioning.

### 2.2 Dependent vs. autonomous cognitive offloading

We draw on the autonomous–dependent help typology (Nadler, 1997, 2015) to distinguish two modes of cognitive offloading. In the helping literature, Nadler (2015)) distinguishes between dependent help—providing ready-made solutions that foster reliance on the helper—and autonomous help—providing tools and guidance that enable recipients to solve problems independently. A recent meta-analysis by Nadler (2015)) confirmed that different help types produce distinct recipient outcomes, underscoring the importance of this distinction. Li et al. (2026)) further demonstrated that receiving dependent help from coworkers harms subsequent creativity by undermining intrinsic motivation, whereas autonomous help enhances creativity by fostering it. We extend this framework from interpersonal helping to human–AI interaction, a move that is increasingly relevant given concerns that AI may function as either a “crutch” or a “scaffold” depending on how it is used (Hsu and Sun, 2025; Carter, 2018).

*Dependent cognitive offloading* occurs when users treat AI as a substitute for their own thinking—accepting AI outputs with minimal evaluation, delegating the structuring of ideas to AI, and relying on AI-generated content as the primary cognitive product. The user's role is reduced to that of a recipient rather than an active thinker.

*Autonomous cognitive offloading*, by contrast, occurs when users treat AI as a scaffold that enhances their own cognitive processes—using AI outputs as starting points for further thinking, comparing AI-generated ideas with one's own reasoning, and maintaining personal ownership of the final cognitive product. The user retains cognitive agency, with AI serving a supportive function.

Because these constructs are introduced here for the first time, it is essential to delineate them from established phenomena in the automation and educational-technology literatures with which they might be confused. We do so along a single organizing principle: dependent and autonomous offloading describe the *task-level manner in which cognitive work is allocated during an AI-assisted episode*, not a global trait, attitude, or competence. Five adjacent constructs warrant explicit comparison. First, *AI dependency*—the habitual, often compulsive reliance on generative AI (Yu et al., 2024)—is a person-level behavioral-addiction syndrome indexed by usage volume, craving, and loss of control; dependent offloading, by contrast, is agnostic to overall usage frequency and concerns only *how* thinking is distributed within a given task. A user could score high on AI dependency yet engage autonomously (using AI heavily but critically), or use AI rarely yet dependently. Second, *automation complacency* and *blind trust* (over-trust) refer to an attentional or attitudinal stance toward an automated agent—reduced monitoring and uncritical acceptance arising from miscalibrated confidence in the tool (Parasuraman and Manzey, 2010); these describe a *disposition toward the tool*, whereas dependent offloading describes an *allocation of the user's own cognitive operations*, and our separate moderator (metacognitive monitoring) captures the monitoring dimension that complacency concerns. Third, *critical AI literacy* is a knowledge-and-skill competence—understanding how AI systems work and how to interrogate their outputs—that is relatively stable across tasks; autonomous offloading is a behavioral enactment that literacy may enable but does not guarantee. Fourth, *self-regulated learning* (SRL) is a broad, domain-general cycle of goal-setting, monitoring, and reflection spanning whole learning episodes; the offloading modes operate at the narrower grain of a single AI-assisted cognitive act and are silent about goal-setting or longer-term regulation. We frame these as conceptual boundaries to be corroborated by future discriminant-validity work against measured instruments (see Appendix A for a construct-boundary summary and Section 5.4).

This distinction is theoretically consequential because the two modes likely involve different levels of cognitive engagement. Dependent offloading may reduce opportunities for deep processing, knowledge construction, and skill development by shifting core cognitive work to AI. Emerging evidence supports this concern: Gerlich 2025) found that ChatGPT-assisted students showed cognitive offloading tendencies that negatively affected learning outcomes, and Fan et al. (2024)) reported that generative AI use reduced self-assessed creative potential. Autonomous offloading, while still leveraging AI's capabilities, preserves the user's active engagement with the material, maintaining the cognitive processes necessary for learning and development (Carter, 2018).

We treat the two offloading modes as qualitatively distinct dimensions rather than opposite poles of a single continuum, consistent with the interpersonal helping literature in which dependent and autonomous help are theorized as separate dimensions that can co-occur (Nadler, 2015; Koopmann, 2016). As we show empirically (Section 4.2), the two scales were only weakly correlated (*r* = 0.08) and a CFA model merging them fit significantly worse than one retaining them as separate factors.

The dependent–autonomous distinction complements rather than replaces content-based classifications of offloading (e.g., by cognitive function offloaded; Risko and Gilbert, 2016). It operates at a different analytical level—capturing *the manner in which* offloading is enacted, regardless of the specific cognitive function involved. We focus on the manner dimension because it is theoretically linked to distinct psychological mechanisms with divergent downstream consequences.

### 2.3 Cognitive agency transfer as a mechanism

We propose *cognitive agency transfer* as a key mechanism linking dependent offloading to poorer perceived subsequent cognitive outcomes. Cognitive agency transfer refers to the process by which individuals progressively cede decision-making authority about cognitive tasks to AI—including decisions about what information is relevant, how to evaluate evidence, and what conclusions to draw. It is important to distinguish this construct from related notions of trust. *Automation trust* and *blind trust* (over-trust) concern a user's *confidence in the tool's accuracy or reliability*, and *automation complacency* concerns *reduced monitoring* of an automated agent; all three are attitudes or attentional states directed *at the tool*. Cognitive agency transfer instead concerns the relocation of *cognitive governance*—who steers the thinking process—from the user to the tool. A user could trust AI's outputs highly yet retain governance (verifying, overriding, and deciding), or distrust them yet still defer governance out of habit or effort-avoidance; trust and the locus of cognitive governance are therefore conceptually separable.

We theorize cognitive agency transfer as a gradual *process* of habituation, but we acknowledge at the outset that the present design measures it as a *state-level self-appraisal* at a single occasion (T2)—that is, the respondent's perceived current degree of deference to AI, which we take to index how far this process has progressed rather than its rate of unfolding. Capturing the process directly would require repeated within-person measurement; we return to this measurement limitation in Section 5.4.

When individuals engage in dependent offloading, they repeatedly experience a pattern in which AI makes core cognitive decisions on their behalf. Over time, this pattern may become internalized: users come to expect AI to handle not only the execution of cognitive tasks but also the *governance* of the cognitive process itself. This concern is consistent with Turner (2022))'s analysis of how digital tool dependency can evolve from cognitive offloading into cognitive “outsourcing,” and with Jose et al. (2025))'s argument that habitual AI delegation produces a gradual erosion of cognitive self-reliance. Dependent offloading should promote cognitive agency transfer because the repeated experience of delegating core thinking to AI gradually normalizes the ceding of cognitive authority (Carter, 2018). In contrast, autonomous offloading, by requiring the user to remain the primary cognitive agent who evaluates, integrates, and ultimately decides, should not produce this transfer.

Hypothesis 1. Dependent cognitive offloading is positively associated with cognitive agency transfer.

Hypothesis 2. Autonomous cognitive offloading is not significantly associated with cognitive agency transfer.

### 2.4 Intrinsic motivation as a mechanism

Self-determination theory (SDT; Deci and Ryan, 1985; Ryan and Deci, 2017) posits that intrinsic motivation—engaging in activities out of interest, enjoyment, and inherent satisfaction—is sustained by the fulfillment of basic psychological needs for autonomy, competence, and relatedness. We argue that the two offloading modes have opposite implications for intrinsic motivation. We note explicitly that the present study measures intrinsic motivation as the *proximal motivational outcome* of SDT but does not measure satisfaction of the autonomy and competence needs themselves. Need satisfaction therefore functions in our account as a theorized intervening mechanism rather than a measured variable; our empirical test concerns its hypothesized motivational consequence, and we treat the underlying need-based mechanism as a proposition for future research that includes need-satisfaction measures (see Section 5.4).

Dependent offloading may threaten both autonomy and competence. When AI makes core cognitive decisions, users experience reduced autonomy because their personal input becomes peripheral to the task outcome. Additionally, dependent offloading may erode perceived competence by implicitly signaling that the user cannot perform the task independently (Nadler, 2015). This is consistent with Chirayath et al. (2025))'s analysis of how AI alters the “mental architecture of coping” through the lens of SDT, showing that AI may enhance competence by offering strategies but simultaneously threaten autonomy by making choices on behalf of users. Autonomous offloading, by contrast, can support both autonomy and competence. By using AI as a scaffold rather than a substitute, users retain meaningful choices about how to approach cognitive tasks (supporting autonomy) and develop their capabilities through effortful engagement (supporting competence). Consistent with Li et al. (2026)), who found that autonomous help enhances recipients' intrinsic motivation while dependent help diminishes it, we predict:

Hypothesis 3. Dependent cognitive offloading is negatively associated with intrinsic motivation.

Hypothesis 4. Autonomous cognitive offloading is positively associated with intrinsic motivation.

### 2.5 Downstream cognitive outcomes

We examine four cognitive outcomes that capture theoretically distinguishable facets of downstream cognitive functioning. *Subsequent autonomous capability* refers to the perceived ability to independently perform similar tasks without AI assistance—it captures *functional self-efficacy* (“Can I do this on my own?”). *Creativity* refers to the generation of novel ideas and approaches—it captures *divergent novelty production* (“Can I produce something original?”). *Deep processing* refers to critical evaluation, integration, and elaboration of information (Craik and Lockhart, 1972)—it captures *analytic elaboration* (“Do I think carefully rather than superficially?”). *Independent judgment* refers to the capacity to form and defend one's own conclusions without external guidance—it captures *epistemic autonomy* (“Can I reach and stand by my own conclusions?”). Although these constructs share a common orientation toward higher-order cognition, they are conceptually distinct: an individual may, for example, retain the ability to work independently (high autonomous capability) while showing reduced originality (lower creativity), or may think deeply about material (high deep processing) while still deferring to others for final conclusions (lower independent judgment). We selected these four outcomes because they span the breadth of cognitive functions most likely to be affected by differential patterns of AI engagement and because they map onto different concerns in the literature: deskilling (Shukla et al., 2025), creativity loss (Fan et al., 2024), shallow processing (Fan et al., 2024), and epistemic dependence (Carter, 2018).

Cognitive agency transfer should be negatively associated with all four outcomes. When individuals have ceded cognitive authority to AI, they have less practice in independent reasoning, reduced confidence in their own judgment, and fewer opportunities to develop the skills underlying creative and analytical thinking (Woodruff et al., 2024; Shukla et al., 2025). Consistent with this logic, Wadinambiarachchi et al. (2024)) found that generative AI use was linked to design fixation and constrained divergent thinking, and Boussioux et al. (2024)) reported differential associations of AI with convergent vs. divergent creative thinking. Intrinsic motivation, conversely, should be positively associated with all four outcomes, as intrinsically motivated individuals engage more deeply with cognitive tasks, persist in the face of difficulty, and explore diverse perspectives (Deci and Ryan, 1985; Ryan and Deci, 2017).

Hypothesis 5. Cognitive agency transfer is negatively associated with (a) subsequent autonomous capability, (b) creativity, (c) deep processing, and (d) independent judgment.

Hypothesis 6. Intrinsic motivation is positively associated with (a) subsequent autonomous capability, (b) creativity, (c) deep processing, and (d) independent judgment.

### 2.6 Dual-pathway mediation

Integrating the above arguments, we propose a dual-pathway mediation model. The indirect effects of dependent offloading on outcomes operate through two parallel channels:

Hypothesis 7a. Dependent cognitive offloading has negative indirect effects on subsequent autonomous capability, creativity, deep processing, and independent judgment via cognitive agency transfer.

Hypothesis 7b. Dependent cognitive offloading has negative indirect effects on subsequent autonomous capability, creativity, deep processing, and independent judgment via reduced intrinsic motivation.

Hypothesis 8. Autonomous cognitive offloading has positive indirect effects on subsequent autonomous capability, creativity, deep processing, and independent judgment via enhanced intrinsic motivation.

**Figure 1:**
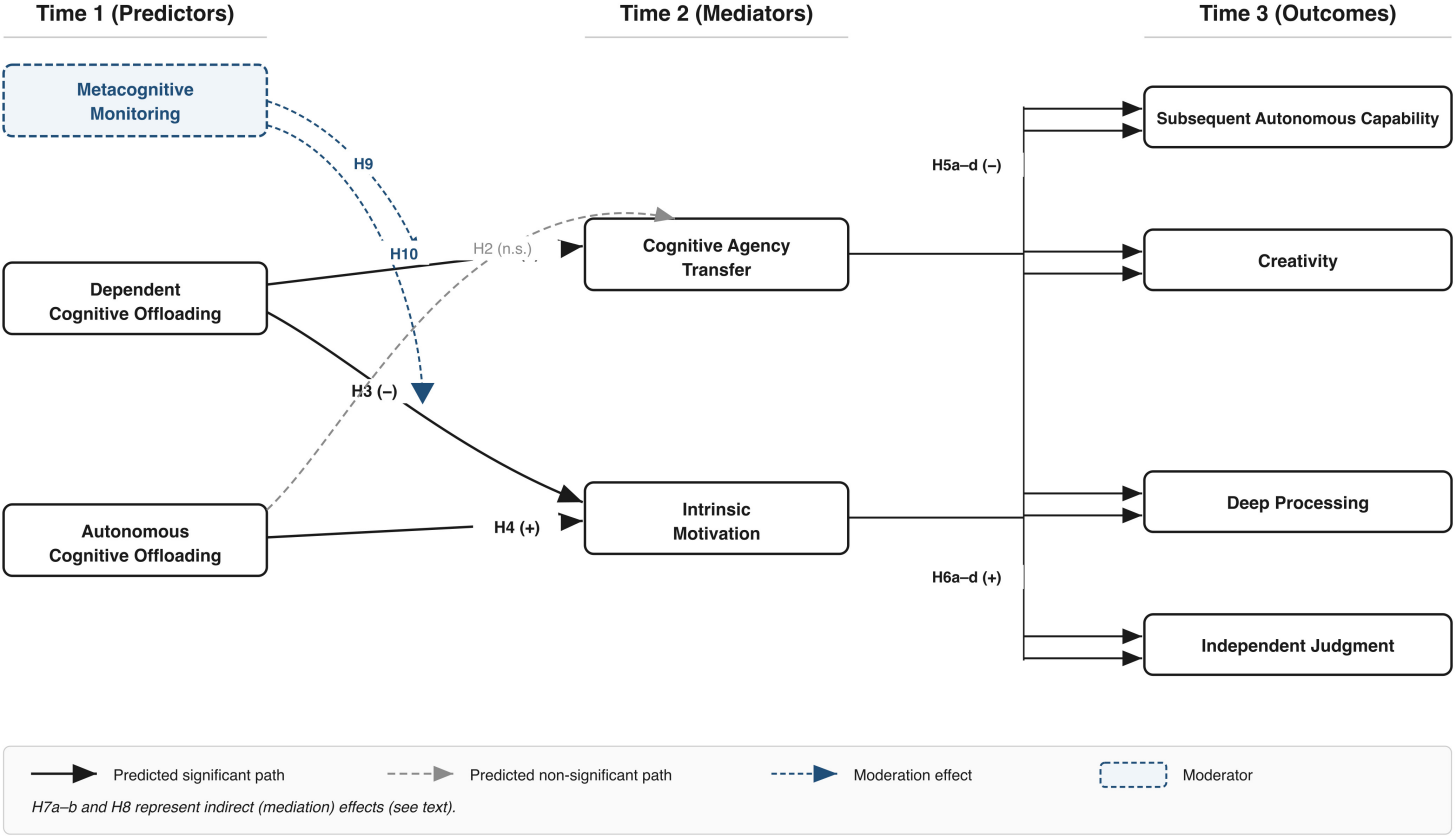
Hypothesized dual-pathway model of cognitive offloading. Solid lines represent predicted significant paths; dashed gray lines represent predicted non-significant paths; dashed blue lines represent hypothesized moderation effects. H7a–b and H8 represent mediation hypotheses (see text).

Because autonomous offloading does not involve ceding cognitive authority to AI (Hypothesis 2), we did not expect significant indirect effects of autonomous offloading on outcomes via cognitive agency transfer. The full hypothesized model is depicted in Figure 1.

### 2.7 The moderating role of metacognitive monitoring

The two pathways traced above express a dialectical tension that is intrinsic to AI-assisted cognition. The same act of offloading can yield *cognitive expansion*—using AI as a scaffold that extends what the user can attempt and understand—or *cognitive disorientation*—a drift in which the user gradually loses the thread of their own reasoning and defers it to the tool. What tips a given episode toward expansion rather than disorientation is, we argue, not the volume of AI use but whether the user keeps the offloading process under deliberate observation. This is precisely the function of metacognition. Generative AI is distinctive in coupling its assistance to an *immediate reward*—fast, fluent, low-effort task completion—that is delivered regardless of whether the user is learning or merely finishing (a point we develop in Section 4.6). Metacognitive monitoring—the capacity to reflect on and regulate one's own cognitive processes (Flavell, 1979; Schraw and Dennison, 1994)—can be understood as a psychological shield against this immediate-reward trap: it is the mechanism that lets a user notice that effortless completion has come at the cost of their own engagement, and intervene before deference becomes habitual. Consistent with this view, Grinschgl et al. (2021)) showed that metacognitive beliefs shape offloading-strategy selection and Boldt and Gilbert (2019)) that metacognitive confidence guides spontaneous offloading, while Turner (2022)) argued that metacognitive awareness is central to preserving intellectual autonomy under pervasive offloading technologies. Susceptibility to the immediate-reward trap is also likely to vary across individuals, making metacognitive monitoring a theoretically motivated boundary condition rather than a uniformly beneficial trait.

Crucially, however, metacognitive monitoring is fundamentally a capacity for *cognitive control*—detecting and correcting how one is thinking—rather than a source of *motivational ownership*. We therefore expect its protective reach to be pathway-specific rather than global. Because cognitive agency transfer is itself a control-related drift (the steady ceding of governance over one's reasoning), it falls squarely within what monitoring can detect and interrupt: a user who notices over-deference can reclaim governance. We thus predict that metacognitive monitoring buffers the dependent-offloading-to- agency-transfer link.

Hypothesis 9. Metacognitive monitoring weakens the positive association between dependent cognitive offloading and cognitive agency transfer.

The motivational pathway is a different matter. The erosion of intrinsic motivation under dependent offloading stems, on the SDT account, from thwarted autonomy and competence experiences—when AI performs the core cognitive work, the user's sense of authorship and challenge is diminished by the structure of the activity itself. Awareness of this diminishment does not restore it; a user can clearly recognize that AI did the meaningful thinking and still experience reduced ownership and interest. Monitoring may even sharpen that recognition. We therefore advance Hypothesis 10 tentatively, expecting any buffering effect on the motivational pathway to be weaker than—and possibly absent relative to—the control pathway, an asymmetry we test directly.

Hypothesis 10. Metacognitive monitoring weakens the negative association between dependent cognitive offloading and intrinsic motivation.

## 3 Materials and methods

### 3.1 Participants and procedure

We conducted a three-wave time-lagged survey study. The study was reviewed and approved by the relevant institutional ethics committee. All participants provided written informed consent before the first wave.

*Recruitment and sample*. Participants were recruited from universities and young professional communities through online survey platforms. Inclusion criteria required that participants (a) were aged 18 or above, (b) had at least 3 months of experience using generative AI tools (e.g., ChatGPT, ERNIE Bot, Kimi, Doubao) for learning or work-related cognitive tasks, and (c) provided informed consent. Participants received modest monetary compensation per completed wave, with a small additional bonus for completing all three waves.

*Three-wave design*. The three survey waves were separated by 2-week intervals. Time 1 (T1) measured independent variables (dependent cognitive offloading, autonomous cognitive offloading), the proposed moderator (metacognitive monitoring), control variables (task complexity, demographics), and AI use background. Time 2 (T2), administered 2 weeks after T1, measured the hypothesized mediators (cognitive agency transfer, intrinsic motivation) and immediate benefit. Time 3 (T3), administered 2 weeks after T2, measured the four outcome variables (subsequent autonomous capability, creativity, deep processing, independent judgment). The 2-week interval was chosen to allow sufficient time for the proposed psychological processes to unfold while minimizing attrition. Participants were matched across waves using unique identifiers generated at T1.

*Sample retention and exclusion*. At T1, 742 participants completed the survey. Of these, 672 (90.6%) completed T2, and 631 (85.0% of T1) completed T3. Each wave included two attention-check items (e.g., “Please select ‘Strongly Agree' for this item”). We also screened for straight-line responding (selecting the same option for all items within a scale) and excessively fast completion (any single wave completed in under 3 min). After matching across all three waves and applying these exclusion criteria—excluding participants who failed attention checks (*n* = 17), exhibited patterned responding (*n* = 14), or completed any wave in under 3 min (*n* = 11)—the final analytic sample comprised 589 participants.

*Sample characteristics*. The final sample (*N* = 589; *M*_age_ = 23.69, *SD* = 3.24; 51.1% female) included undergraduate students (54.3%), master's students (25.3%), doctoral students (8.0%), and young professionals (12.4%). Regarding AI use frequency, 14.1% reported using AI rarely, 20.4% occasionally, 26.5% sometimes, 23.6% frequently, and 15.4% very frequently. The mean duration of generative AI use experience was approximately 12 months. A comparison of participants who completed all three waves vs. those who dropped out revealed no significant differences in gender (χ^2^ = 0.91, *p* = 0.34), age (*t* = 1.08, *p* = 0.28), education level (χ^2^ = 2.45, *p* = 0.49), or T1 dependent offloading (*t* = 0.72, *p* = 0.47), suggesting that attrition was not systematically related to key study variables.

### 3.2 Measures

All scales were measured on 5-point Likert scales (1 = *strongly disagree*; 5 = *strongly agree*). Because existing instruments do not directly capture AI-specific offloading modes or cognitive agency transfer, we developed theory-grounded, context-specific measures drawing on the most theoretically relevant prior scales and adapting items for the generative AI context. We followed a streamlined scale-adaptation procedure designed to preserve content validity while keeping the instrument feasible for a multi-wave design, and translated all measures into Chinese using Brislin (1986)) back-translation. Item development involved three stages: (1) drafting 6–8 items per construct based on theoretical definitions and existing scales; (2) expert review by four subject-matter experts in educational psychology, organizational behavior, and human–AI interaction, who rated item relevance and clarity; and (3) pilot testing with 30 graduate students to identify ambiguous wording and task-context mismatches. Items with overlapping wording or unclear referents were revised or removed before the final survey. This procedure yielded 4 items per construct. We emphasize that these are *preliminary, domain-specific instruments*: the present evidence establishes internal consistency, convergent validity, and discriminant validity among the study constructs (Section 4.1), but full psychometric validation—including discriminant validity against measured adjacent constructs (e.g., AI dependency, critical AI literacy) and test–retest stability—remains for future work. Complete item wordings are provided in Appendix B.

#### 3.2.1 Time 1 measures

*Dependent cognitive offloading* (4 items). Because no existing measure captures dependent cognitive offloading in the generative AI context, we developed items drawing on two sources: (1) the cognitive offloading framework (Risko and Gilbert, 2016), which defines offloading as delegating cognitive demands to external tools, and (2) the dependent help scale (Koopmann, 2016), which operationalizes help-seeking that fosters reliance on the helper. Items were rewritten to describe AI-specific behaviors in which users delegate core thinking to AI rather than engaging with the material themselves. A sample item is “When using AI for a task, I prefer to let AI generate the complete answer directly” [α = 0.773; composite reliability [CR] = 0.85; average variance extracted [AVE] = 0.59; corrected item–total correlations [CITC] ranged from 0.53 to 0.62].

*Autonomous cognitive offloading* (4 items). Drawing on the autonomous help scale (Koopmann, 2016), we developed items capturing AI use in which the user retains cognitive agency—using AI outputs as inputs for further thinking rather than as final products. A sample item is “When using AI for a task, I use AI suggestions as a starting point for my own thinking” (α = 0.767; CR = 0.85; AVE = 0.59; CITC: 0.52–0.61).

*Metacognitive monitoring* (4 items). Items were adapted from the metacognitive awareness inventory (Schraw and Dennison, 1994) to capture awareness and regulation during AI use. A sample item is “While using AI, I regularly check whether I truly understand the content rather than just accepting it” (α = 0.765; CR = 0.85; AVE = 0.59; CITC: 0.53–0.61).

*Task complexity* (4 items). Items assessed the cognitive demands of tasks for which participants typically used AI (Kalyuga, 2007). A sample item is “The tasks for which I use AI often require integrating multiple perspectives” (α = 0.760; CR = 0.85; AVE = 0.58; CITC: 0.50–0.61).

*Control variables*. We controlled for gender (1 = male, 2 = female), age, education level (1 = undergraduate, 2 = master's, 3 = doctoral, 4 = working professional), AI use frequency (1 = rarely to 5 = very frequently), and AI use experience (months).

#### 3.2.2 Time 2 measures

*Cognitive agency transfer* (4 items). Because this construct is novel to the human–AI interaction literature and no existing measure captures it, we developed items directly from the theoretical definition: the progressive ceding of cognitive decision-making authority—about relevance, evaluation, and conclusion formation—to AI. A sample item is “I increasingly rely on AI to determine which information is most important for my tasks” (α = 0.773; CR = 0.86; AVE = 0.60; CITC: 0.52–0.63).

*Intrinsic motivation* (4 items). Items were adapted from Shin and Zhou's (2003)) scale, modified for the AI use context. A sample item is “I feel genuinely interested in thinking through the problems I work on, even when AI could do it for me” (α = 0.837; CR = 0.89; AVE = 0.67; CITC: 0.63–0.71).

*Immediate benefit* (4 items). Items captured perceived efficiency gains from AI use. A sample item is “Using AI allows me to complete tasks faster and with less effort” (α = 0.750; CR = 0.84; AVE = 0.58; CITC: 0.51–0.60).

#### 3.2.3 Time 3 measures

*Subsequent autonomous capability* (4 items). Items assessed confidence and ability to perform similar tasks independently without AI, adapted from Nadler (2015)). A sample item is “I feel confident that I can handle similar tasks on my own without AI” (α = 0.793; CR = 0.87; AVE = 0.62; CITC: 0.57–0.65).

*Creativity* (4 items). Items were adapted from Zhou and George (2001)) for the AI use domain. A sample item is “I can generate novel ideas and approaches when working on tasks in this domain” (α = 0.747; CR = 0.84; AVE = 0.57; CITC: 0.48–0.60).

*Deep processing* (4 items). Items captured critical evaluation and elaboration of information (Craik and Lockhart, 1972). A sample item is “I carefully evaluate and think deeply about information rather than accepting it at face value” (α = 0.848; CR = 0.90; AVE = 0.69; CITC: 0.65–0.71).

*Independent judgment* (4 items). Items assessed the ability to form conclusions without external support. A sample item is “I can independently evaluate information and reach well-reasoned conclusions” (α = 0.746; CR = 0.84; AVE = 0.57; CITC: 0.50–0.58).

### 3.3 Analytical strategy

We tested our hypotheses in Mplus 8.3 using maximum likelihood estimation. The CFA was estimated at the item level, with each construct represented by its four observed indicators. Missing item responses in the item-level CFA were handled with maximum likelihood estimation under the missing-at-random assumption. After establishing the measurement model, we estimated the hypothesized structural paths. The primary structural model used manifest (mean) scale scores for T1 predictors, T2 mediators, and T3 outcomes; we chose this specification for parsimony and stable estimation of the full mediated–moderated model (which involves 11 constructs, a latent interaction, and six covariates) at the available sample size. Because manifest scores do not correct for measurement error, we re-estimated the core mediation structure as a fully latent-variable SEM as a robustness check (Section 4.7), and we verified that the conclusions held across theoretically distinct subsamples (students vs. early-career workers; Section 4.7). For the structural path analyses, participants were retained when the relevant scale scores were available. Indirect effects were assessed using bias-corrected bootstrapping with 10,000 replications (Preacher and Hayes, 2008). For moderation hypotheses, we created interaction terms between mean-centered dependent offloading and mean-centered metacognitive monitoring. Model fit was evaluated using standard criteria: CFI ≥ 0.90, TLI ≥ 0.90, RMSEA ≤ 0.08, and SRMR ≤ 0.08 (Hu and Bentler, 1999).

## 4 Results

### 4.1 Measurement model

#### 4.1.1 Confirmatory factor analysis

We first assessed the measurement model through CFA. The hypothesized 11-factor model (dependent offloading, autonomous offloading, metacognitive monitoring, task complexity, cognitive agency transfer, intrinsic motivation, immediate benefit, subsequent autonomous capability, creativity, deep processing, and independent judgment) showed acceptable fit: χ^2^(847) = 1825.22, CFI = 0.92, TLI = 0.91, RMSEA = 0.044, SRMR = 0.042. All standardized factor loadings were significant and ranged from 0.72 to 0.85 (mean = 0.78), exceeding the 0.50 threshold.

To establish discriminant validity, we compared the hypothesized model against several theoretically meaningful alternatives (Table 1). The 11-factor model fit significantly better than a 10-factor model merging dependent and autonomous offloading (Δχ^2^ = 726.53, Δ*df* = 10, *p* < 0.001), supporting the treatment of these as distinct constructs. It also fit significantly better than a 10-factor model merging cognitive agency transfer and intrinsic motivation (Δχ^2^ = 1354.54, Δ*df* = 10, *p* < 0.001), supporting the distinctiveness of the two mediators. An 8-factor model merging all four T3 outcomes into a single factor showed substantially worse fit (Δχ^2^ = 1198.87, Δ*df* = 27, *p* < 0.001), supporting the treatment of the four cognitive outcomes as separate constructs. Finally, a 1-factor model fit poorly (Δχ^2^ = 4690.36, *p* < 0.001), suggesting that the observed pattern is unlikely to be an artifact of a single underlying method factor, although this test does not rule out shared method variance entirely.

**Table 1:** Confirmatory factor analysis: model comparisons.

Model	χ^2^	*df*	CFI	TLI	RMSEA	*Δχ* ^2^	*Δdf*
11-factor (hypothesized)	1825.22	847	0.92	0.91	0.044	–	–
10-factor (DO + AO merged)	2551.75	857	0.86	0.84	0.058	726.53^***^	10
10-factor (CAT + IM merged)	3179.76	857	0.82	0.80	0.067	1354.54^***^	10
8-factor (T3 outcomes merged)	3024.09	874	0.83	0.81	0.064	1198.87^***^	27
1-factor	6515.58	902	0.51	0.47	0.102	4690.36^***^	55

*N* = 589. DO, dependent offloading; AO, autonomous offloading; CAT, cognitive agency transfer; IM, intrinsic motivation. ^***^p < 0.001.

#### 4.1.2 Reliability and convergent validity

Table 2 summarizes the reliability and convergent validity indices. All Cronbach's α values ranged from 0.746 to 0.848, exceeding the .70 threshold (Nunnally and Bernstein, 1994). All composite reliability (CR) values exceeded 0.84, and all average variance extracted (AVE) values exceeded .57, surpassing the respective .70 and .50 benchmarks (Fornell and Larcker, 1981). Corrected item–total correlations for all items exceeded .49.

**Table 2:** Reliability and convergent validity of study measures.

Time	Scale	Items	α	CR	AVE	$\sqrt{\text{AVE}}$	CITC range
T1	Dependent offloading	4	0.773	0.85	0.59	0.77	0.53–0.62
	Autonomous offloading	4	0.767	0.85	0.59	0.77	0.52–0.61
	Metacognitive monitoring	4	0.765	0.85	0.59	0.77	0.53–0.61
	Task complexity	4	0.760	0.85	0.58	0.76	0.50–0.61
T2	Cognitive agency transfer	4	0.773	0.85	0.60	0.77	0.52–0.63
	Intrinsic motivation	4	0.837	0.89	0.67	0.82	0.63–0.71
	Immediate benefit	4	0.750	0.84	0.58	0.76	0.51–0.60
T3	Subsequent autonomous capability	4	0.793	0.87	0.62	0.79	0.57–0.65
	Creativity	4	0.747	0.84	0.57	0.76	0.48–0.60
	Deep processing	4	0.848	0.90	0.69	0.83	0.65–0.71
	Independent judgment	4	0.746	0.84	0.57	0.75	0.50–0.58

*N* = 589. α, Cronbach's alpha; CR, composite reliability; AVE, average variance extracted; CITC, corrected item–total correlation.

#### 4.1.3 Discriminant validity

Discriminant validity was further evaluated using the Fornell–Larcker criterion: for every pair of constructs, the square root of each construct's AVE exceeded all inter-construct correlations (see Table 3), confirming that each construct was more strongly related to its own indicators than to any other construct.

**Table 3:** Means, standard deviations, and correlations among study variables.

	Variable	*M*	*SD*	1	2	3	4	5	6	7	8	9	10	11
1.	Dep. offloading (T1)	3.15	1.01	**0.77**										
2.	Aut. offloading (T1)	3.21	0.98	0.08^*^	**0.77**									
3.	Metacog. monitoring (T1)	3.24	0.98	−0.13^**^	0.15^**^	**0.77**								
4.	Task complexity (T1)	3.12	1.00	0.26^**^	0.19^**^	0.03	**0.76**							
5.	Cog. agency transfer (T2)	2.91	1.00	0.38^**^	−0.01	−0.12^**^	0.19^**^	**0.77**						
6.	Intrinsic motivation (T2)	3.34	1.07	−0.19^**^	0.29^**^	0.35^**^	0.05	−0.11^**^	**0.82**					
7.	Immediate benefit (T2)	3.49	0.94	0.22^**^	0.22^**^	0.06	0.27^**^	0.10^*^	0.13^**^	**0.76**				
8.	Subseq. aut. capability (T3)	3.17	1.01	−0.23^**^	0.20^**^	0.33^**^	−0.02	−0.25^**^	0.39^**^	0.06	**0.79**			
9.	Creativity (T3)	3.25	0.96	−0.13^**^	0.21^**^	0.20^**^	0.06	−0.17^**^	0.28^**^	0.12^**^	0.22^**^	**0.75**		
10.	Deep processing (T3)	3.20	1.07	−0.16^**^	0.28^**^	0.29^**^	0.09^*^	−0.23^**^	0.39^**^	0.08^*^	0.29^**^	0.25^**^	**0.83**	
11.	Indep. judgment (T3)	3.05	0.96	−0.30^**^	0.14^**^	0.25^**^	−0.01	−0.32^**^	0.34^**^	−0.00	0.28^**^	0.16^**^	0.23^**^	**0.75**

*N* = 589. ^*^p < 0.05; ^**^p < 0.01. Values in bold on the diagonal are $\sqrt{\text{AVE}}$ for each construct.

### 4.2 Descriptive statistics and correlations

Table 3 presents the means, standard deviations, and zero-order correlations among all study variables. The correlation patterns were consistent with the proposed theoretical model. Dependent offloading was positively correlated with cognitive agency transfer (*r* = 0.38, *p* < 0.001) and negatively correlated with intrinsic motivation (*r* = −0.19, *p* < 0.001). Autonomous offloading showed a non-significant correlation with cognitive agency transfer (*r* = −0.01, *p* = 0.83) but a positive correlation with intrinsic motivation (*r* = 0.29, *p* < 0.001). Importantly, the two offloading modes were only weakly correlated with each other (*r* = 0.08, *p* = 0.049), suggesting they represent largely independent dimensions rather than opposite poles of a single continuum.

Cognitive agency transfer was negatively correlated with all four T3 outcomes (*rs* from −0.17 to −0.32, all *ps* < 0.001), and intrinsic motivation was positively correlated with all four T3 outcomes (*rs* from 0.28 to 0.39, all *ps* < 0.001).

### 4.3 Structural model: direct effects

**Figure 2:**
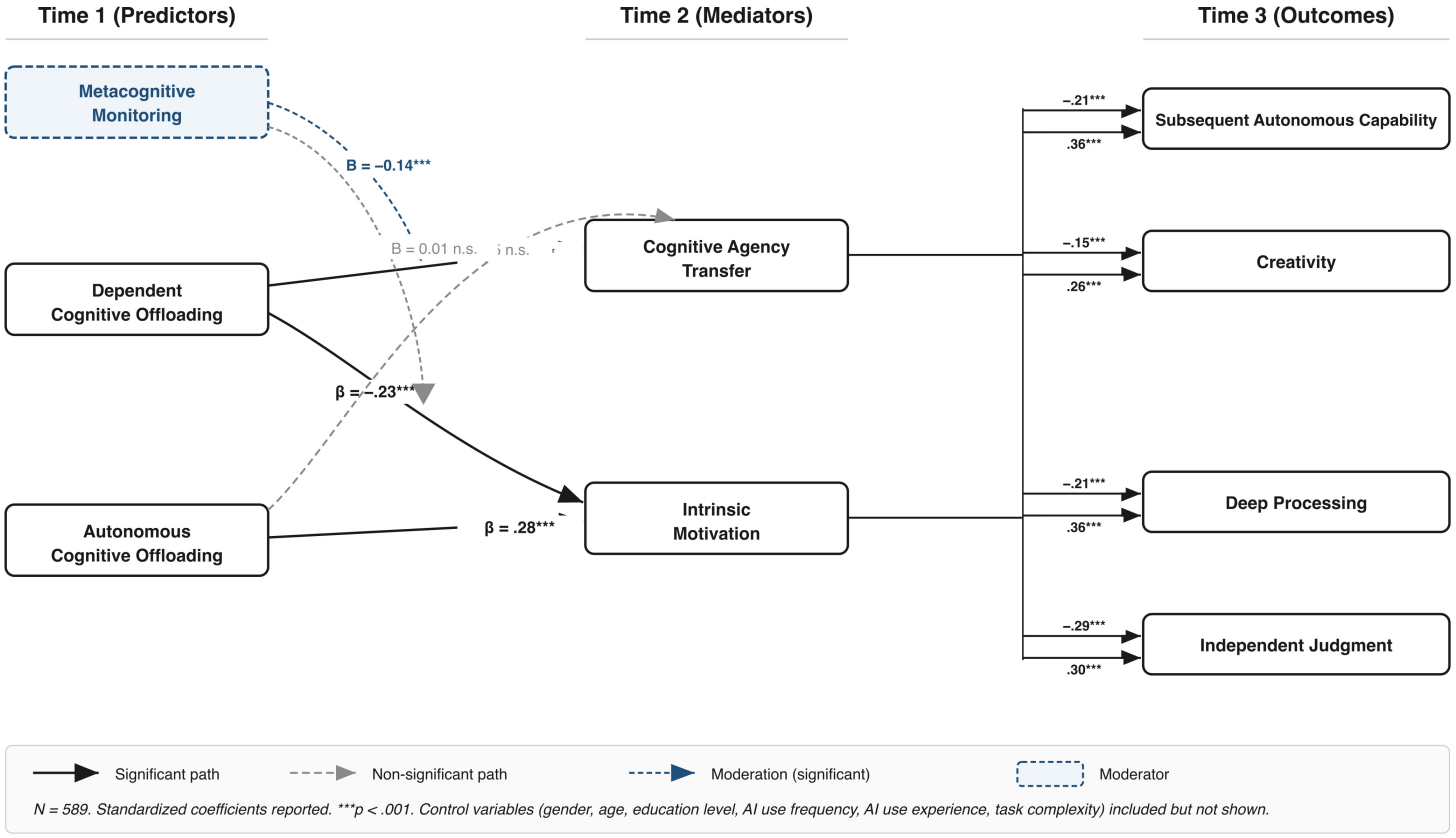
Structural model results with standardized path coefficients. Solid lines represent significant paths; dashed gray lines represent non-significant paths; dashed blue lines represent moderation effects. ^***^p < 0.001, ^**^p < 0.01. *N* = 589. Control variables (gender, age, education level, AI use frequency, AI use experience, and task complexity) included but not shown.

The structural model with T1 predictors, T2 mediators, and T3 outcomes (controlling for gender, age, education level, AI use frequency, AI use experience, and task complexity; see Figure 2 for an overview) demonstrated good fit: χ^2^(52) = 138.47, CFI = 0.96, TLI = 0.93, RMSEA = 0.053, SRMR = 0.038.

#### 4.3.1 Effects on mediators

Consistent with Hypothesis 1, dependent offloading was positively associated with cognitive agency transfer (*B* = 0.35, *SE* = 0.04, *p* < 0.001, β = 0.35). Consistent with Hypothesis 2, autonomous offloading was not significantly associated with cognitive agency transfer (*B* = −0.05, *SE* = 0.04, *p* = 0.18, β = −0.05). Supporting Hypothesis 3, dependent offloading was negatively associated with intrinsic motivation (*B* = −0.25, *SE* = 0.04, *p* < 0.001, β = −0.23). Supporting Hypothesis 4, autonomous offloading was positively associated with intrinsic motivation (*B* = 0.30, *SE* = 0.04, *p* < 0.001, β = 0.28).

#### 4.3.2 Effects on outcomes

Table 4 presents the path coefficients from T2 mediators to T3 outcomes. Consistent with Hypothesis 5 (a–d), cognitive agency transfer was negatively associated with all four outcomes. Consistent with Hypothesis 6 (a–d), intrinsic motivation was positively associated with all four outcomes. The associations involving intrinsic motivation were notably stronger and more consistent across outcomes (standardized βs from 0.26 to 0.36) than those involving cognitive agency transfer (βs from −0.15 to −0.29), suggesting that the motivational pathway may be the more potent channel linking offloading modes to subsequent cognition.

**Table 4:** Structural model: effects of T2 mediators on T3 cognitive outcomes.

	Subsequent capability		Creativity		Deep processing		Independent judgment	
Predictor	β	*p*	β	*p*	β	*p*	β	*p*
Cog. agency transfer	−0.21	<0.001	−0.15	<0.001	−0.21	<0.001	−0.29	<0.001
Intrinsic motivation	0.36	<0.001	0.26	<0.001	0.36	<0.001	0.30	<0.001
*R* ^2^	0.20		0.11		0.20		0.20	

*N* = 589. Standardized coefficients reported. Controls (gender, age, education level, AI use frequency, AI use experience, and task complexity) included but not shown.

### 4.4 Mediation: indirect effects

Table 5 reports the bootstrapped indirect effects. Supporting Hypothesis 7a, the indirect effects of dependent offloading through cognitive agency transfer were significant for all four outcomes: subsequent autonomous capability [β = −0.07, 95% CI [−0.11, −0.04]], creativity [β = −0.05, 95% CI [−0.09, −0.02]], deep processing [β = −0.07, 95% CI [−0.11, −0.04]], and independent judgment [β = −0.10, 95% CI [−0.14, −0.07]]. Supporting Hypothesis 7b, the indirect effects through intrinsic motivation were also significant: subsequent autonomous capability [β = −0.09, 95% CI [−0.12, −0.05]], creativity [β = −0.06, 95% CI [−0.09, −0.03]], deep processing [β = −0.08, 95% CI [−0.12, −0.05]], and independent judgment [β = −0.07, 95% CI [−0.11, −0.04]].

**Table 5:** Bootstrapped indirect effects of cognitive offloading on T3 outcomes via T2 mediators.

IV	Outcome	Mediator	β	95% CI
Dep. offloading	Subseq. aut. capability	CAT	−0.07	[−0.11, −0.04]
	Subseq. aut. capability	IM	−0.09	[−0.12, −0.05]
	Creativity	CAT	−0.05	[−0.09, −0.02]
	Creativity	IM	−0.06	[−0.09, −0.03]
	Deep processing	CAT	−0.07	[−0.11, −0.04]
	Deep processing	IM	−0.08	[−0.12, −0.05]
	Indep. judgment	CAT	−0.10	[−0.14, −0.07]
	Indep. judgment	IM	−0.07	[−0.11, −0.04]
Aut. offloading	Subseq. aut. capability	IM	0.10	[0.07, 0.14]
	Creativity	IM	0.07	[0.04, 0.10]
	Deep processing	IM	0.10	[0.07, 0.14]
	Indep. judgment	IM	0.08	[0.05, 0.12]

*N* = 589. Standardized indirect effects with bias-corrected 95% CIs from 10,000 bootstrap replications. CAT, cognitive agency transfer; IM, intrinsic motivation; IV, independent variable.

Supporting Hypothesis 8, autonomous offloading showed significant positive indirect effects on all four outcomes through intrinsic motivation: subsequent autonomous capability [β = 0.10, 95% CI [0.07, 0.14]], creativity [β = 0.07, 95% CI [0.04, 0.10]], deep processing [β = 0.10, 95% CI [0.07, 0.14]], and independent judgment [β = 0.08, 95% CI [0.05, 0.12]]. As expected, the indirect effects of autonomous offloading through cognitive agency transfer were not significant (all 95% CIs included zero).

### 4.5 Moderation: metacognitive monitoring

To test Hypotheses 9 and 10, we added the interaction term of mean-centered dependent offloading × mean-centered metacognitive monitoring as a predictor of both T2 mediators.

Supporting Hypothesis 9, the interaction significantly predicted cognitive agency transfer (*B* = −0.14, *SE* = 0.04, *p* < 0.001). Simple slope analysis revealed that the positive association between dependent offloading and cognitive agency transfer was stronger when metacognitive monitoring was low (−1 *SD*: β = 0.48, *p* < 0.001) than when it was high (+1 *SD*: β = 0.21, *p* < 0.001). Importantly, dependent offloading remained a significant predictor even at high metacognitive monitoring, indicating that metacognitive awareness attenuates but does not eliminate the association.

Hypothesis 10, however, was not supported. The interaction between dependent offloading and metacognitive monitoring did not significantly predict intrinsic motivation (*B* = 0.01, *SE* = 0.04, *p* = 0.72). The negative association between dependent offloading and intrinsic motivation was comparable at low metacognitive monitoring (−1 *SD*: β = −0.19, *p* < 0.001) and high metacognitive monitoring (+1 *SD*: β = −0.17, *p* < 0.001), suggesting that metacognitive awareness does not buffer the motivational costs of dependent offloading.

### 4.6 Additional analysis: the role of immediate benefit

Immediate benefit (T2) was positively correlated with both dependent offloading (*r* = 0.22) and autonomous offloading (*r* = 0.22, both *ps* < 0.001), indicating that both offloading modes are associated with comparable short-term perceived gains. However, immediate benefit showed weak and largely non-significant associations with T3 cognitive outcomes (subsequent autonomous capability: *r* = 0.06, *p* = 0.14; independent judgment: *r* = −0.00, *p* = 0.91). This pattern is consistent with the possibility that immediate utility does not differentiate between offloading strategies that are associated with more vs. less favorable downstream correlates. We return to the interpretation of this dissociation in the Discussion, where we are careful not to over-read it.

### 4.7 Robustness checks

#### 4.7.1 Latent-variable specification

Because the primary structural model used manifest scale scores, which do not correct for measurement error, we re-estimated the core mediation structure as a fully latent-variable SEM, with each of the ten substantive constructs represented by its four indicators and the same set of covariates. The latent model fit the data well, χ^2^(891) = 938.85, CFI = 0.99, TLI = 0.99, RMSEA = 0.010. As shown in Table 6, all structural conclusions were reproduced: every path retained its sign and significance status, dependent offloading remained positively associated with cognitive agency transfer and autonomous offloading unrelated to it, the two predictors retained their opposite associations with intrinsic motivation, and the two mediators retained their opposite associations with all four outcomes. The latent coefficients were comparable to, and in several cases slightly larger than, their manifest-score counterparts—the expected consequence of correcting for attenuation due to measurement error—indicating that the manifest-score estimates were, if anything, conservative.

**Table 6:** Manifest- vs. latent-variable structural estimates (standardized).

Path	Manifest β	Latent β
T1 predictors→T2 mediators		
Dep. offloading → cognitive agency transfer	0.35^***^	0.43^***^
Aut. offloading → cognitive agency transfer	−0.05	−0.06
Dep. offloading → intrinsic motivation	−0.23^***^	−0.22^***^
Aut. offloading → intrinsic motivation	0.28^***^	0.27^***^
T2 mediators→T3 outcomes		
Cognitive agency transfer → subseq. aut. capability	−0.21^***^	−0.24^***^
Intrinsic motivation → subseq. aut. capability	0.36^***^	0.40^***^
Cognitive agency transfer → creativity	−0.15^***^	−0.17^**^
Intrinsic motivation → creativity	0.26^***^	0.26^***^
Cognitive agency transfer → deep processing	−0.21^***^	−0.24^***^
Intrinsic motivation → deep processing	0.36^***^	0.35^***^
Cognitive agency transfer → indep. judgment	−0.29^***^	−0.29^***^
Intrinsic motivation → indep. judgment	0.30^***^	0.30^***^

*N* = 589. Standardized coefficients. The manifest column reproduces the primary structural model (Sections 4.3.1–4.3.2); the latent column is the fully latent-variable SEM (each construct measured by four indicators), which fit the data well, χ^2^(891) = 938.85, CFI = 0.99, TLI = 0.99, RMSEA = 0.010. Both models include gender, age, education level, AI use frequency, AI use experience, and task complexity as covariates. ^**^p < 0.01; ^***^p < 0.001.

#### 4.7.2 Students vs. early-career workers

To assess whether combining university students (*n* = 516) with early-career workers (*n* = 73) obscured group differences, we re-estimated the key paths separately in each subgroup. The overall pattern held in both: dependent offloading was positively associated with cognitive agency transfer (students β = 0.40; workers β = 0.30; both *p* ≤ 0.011), autonomous offloading was unrelated to it in both groups, and the two mediators showed the expected opposite associations with the four outcomes in both groups (all signs consistent). As expected given the small worker subsample, a few worker-group coefficients did not reach significance (e.g., cognitive agency transfer to subsequent autonomous capability, β = −0.15, *p* = 0.16), but none reversed direction. Formal moderation tests (group × dependent offloading) were non-significant for the cognitive agency transfer pathway (β = −0.10, *p* = 0.36) but significant for the motivational pathway (β = −0.26, *p* = 0.022): the negative association between dependent offloading and intrinsic motivation was somewhat stronger among workers (β = −0.47) than students (β = −0.18). We interpret this single group difference cautiously given the modest worker sample, but it suggests that the motivational cost of dependent offloading may be at least as pronounced outside educational settings—an issue we flag for future, adequately powered comparisons.

## 5 Discussion

Using a three-wave time-lagged design, this study provides correlational evidence that AI offloading mode is linked to downstream perceived cognitive functioning through distinct agency-related and motivational pathways. The findings form a coherent pattern rather than a collection of isolated significant effects: dependent offloading was associated with greater ceding of cognitive authority and lower intrinsic motivation, whereas autonomous offloading was associated primarily with preserved motivation without elevated agency transfer. Metacognitive monitoring attenuated the cognitive agency transfer pathway but did not buffer the negative motivational association of dependent offloading. Below, we discuss how these findings extend prior work and outline their implications.

### 5.1 Contributions and implications for theory

#### 5.1.1 A mode-based distinction in cognitive offloading

The present findings extend prior work by showing that offloading to generative AI is not adequately captured by a unidimensional account. Two individuals who use AI equally frequently may show divergent cognitive correlates depending on *how* they engage with AI (cf. Risko and Gilbert, 2016; Carter, 2018). The two offloading modes were only weakly correlated (*r* = 0.08), supporting their treatment as nearly orthogonal dimensions that can co-occur within the same individual—suggesting a 2 × 2 space of AI engagement profiles that a unidimensional model cannot capture. Future research using person-centered methods (e.g., latent profile analysis) could identify these profiles and their differential associations with cognitive outcomes. We note that establishing discriminant validity against adjacent constructs (e.g., AI dependency, automation trust) remains a priority for future measurement work.

#### 5.1.2 A dual-pathway mechanism with asymmetric moderation

A second contribution is the identification of cognitive agency transfer and intrinsic motivation as parallel mechanisms linking offloading modes to divergent downstream correlates. The consistent pattern across four outcome variables suggests that these mechanisms operate broadly across facets of perceived cognitive functioning. Notably, the cognitive agency transfer pathway has no direct parallel in the interpersonal helping literature: while Li et al. (2026)) showed that dependent help undermines creativity through reduced intrinsic motivation, the human–AI context introduces the possibility of transferring cognitive *authority* to the tool—a deeper form of dependence consistent with concerns about AI-induced deskilling (Shukla et al., 2025; Jose et al., 2025). We emphasize that our survey design provides evidence of association, not causation.

The moderation findings reveal an informative asymmetry. Metacognitive monitoring attenuated the association between dependent offloading and cognitive agency transfer (β = 0.48 at low monitoring vs. β = 0.21 at high monitoring) but did not buffer the negative motivational association. This suggests that self-monitoring protects *cognitive control*—helping users recognize overreliance—but not *motivational ownership*: when AI completes core cognitive work, the user's sense of autonomous engagement and competence may diminish regardless of awareness. This asymmetry implies that structural interventions in AI tool design may be needed alongside metacognitive training (Hsu and Sun, 2025; Carter, 2018).

The divergence was especially visible in pathways to creativity, where direct associations were modest but indirect effects through both mechanisms were consistent—suggesting that creativity is shaped less by AI use *per se* than by whether AI use preserves intrinsic engagement and cognitive ownership (cf. Li et al., 2026).

The mode-based framework generates testable predictions that a unidimensional account does not: (a) *manner* of AI use should predict downstream correlates more strongly than *frequency*; (b) metacognitive monitoring should buffer agency transfer but not motivational loss; and (c) immediate benefits should fail to distinguish adaptive from maladaptive use. Whether cognitive agency transfer is domain-specific or generalizes across task types remains an open question for future research.

#### 5.1.3 Immediate utility vs. downstream cognitive correlates

The additional analysis reveals a noteworthy dissociation: both offloading modes showed equivalent associations with immediate benefit (*r*s of 0.22), yet opposite patterns of association with subsequent perceived cognitive outcomes. We advance the following interpretation tentatively, as our design tests neither users' detection of these patterns nor the persistence of their behavior over time. Because dependent and autonomous offloading were reported as similarly productive in the moment, the present data provide no evidence that the immediate experience itself signals which strategy carries less favorable downstream correlates. One possibility—which we frame as a hypothesis for future work rather than an established finding—is that immediate benefit functions as a *reinforcement signal* sustaining whichever offloading pattern a user has adopted, a dynamic analogous to the “competency trap” in organizational learning, in which positive performance feedback discourages exploration of superior strategies (Kim et al., 2026). On this reading, immediate benefit would be not merely a control variable but a theoretically important factor; testing it, however, would require measuring users' in-the-moment appraisals of strategy quality and tracking behavioral change, which the present design does not do.

### 5.2 Methodological considerations

Two design choices warrant justification. First, all outcome variables were self-reported. We note that the constructs of interest—subsequent autonomous capability, creativity, deep processing, and independent judgment—capture subjective appraisals of one's own cognitive functioning, which are theoretically meaningful in their own right: individuals' beliefs about their cognitive capacity shape subsequent engagement strategies, help-seeking behavior, and willingness to attempt challenging tasks independently (Nadler, 2015). The present findings should therefore be interpreted as pertaining to *perceived* rather than *demonstrated* cognitive outcomes; the implications of this boundary are discussed further in the Limitations.

Second, participants reported on their offloading behavior across the range of tasks for which they typically use AI, rather than within a single standardized task. This task heterogeneity increases ecological validity but introduces uncontrolled variance, as different task types may elicit different offloading patterns. Whether the dual-pathway model operates consistently across task types remains an open question for future research.

### 5.3 Practical implications

Given that our sample consisted primarily of university students and early-career knowledge workers, the practical implications we discuss are most directly applicable to educational and early-professional contexts.

For *educators*, the results suggest that the focus should shift from whether students use AI to how they use it. Educational interventions should teach students to use AI as a scaffold—prompting AI for alternative perspectives, critically evaluating AI outputs, and maintaining ownership of their cognitive process—while explicitly discouraging the wholesale delegation of thinking to AI. Metacognitive training may help individuals resist ceding cognitive authority to AI, but our results suggest it does not attenuate the negative motivational association of dependent offloading. This asymmetry challenges the prevailing assumption in educational technology that fostering metacognitive awareness is a sufficient safeguard against the risks of AI-assisted learning (Hsu and Sun, 2025); it suggests that motivational design—structuring tasks so that students experience genuine ownership and challenge even when AI is available—may be equally important.

For *organizations*, the results highlight that short-term efficiency gains from AI use may not translate into sustained cognitive development. Training programs that promote autonomous AI engagement and metacognitive monitoring may help organizations realize AI's benefits while reducing the risk of the unfavorable cognitive correlates identified here.

For *AI tool designers*, the findings suggest value in designing features that encourage autonomous rather than dependent offloading—for example, by providing explanations alongside suggestions, prompting users to evaluate AI outputs before accepting them, and scaffolding metacognitive reflection. Given that users cannot distinguish beneficial from maladaptive engagement based on immediate experience alone, tool-level interventions (e.g., friction points that prompt reflection before accepting AI outputs, task-sensitive scaffolding that varies by cognitive demand) may be especially important.

### 5.4 Limitations and future directions

Several limitations should be noted, and they bound our claims in important ways. First, and most fundamentally, all outcomes were *self-reported appraisals of cognitive functioning*, not performance-based measures. Our findings therefore speak to associations among *perceived* autonomous capability, creativity, deep processing, and independent judgment—not to demonstrated changes in actual cognitive ability. We have used associational language throughout for this reason, and the results should not be read as evidence that AI offloading improves or impairs cognitive capacity itself. Self-reported and demonstrated cognition can diverge, and establishing whether the present associations extend to behavioral performance is a priority for future work using unaided task performance and AI-interaction logs. Relatedly, because several core constructs used newly developed scales, common method variance is a concern; the temporal separation between waves and the poor fit of a single-factor model (CFI = .51)—expected if responses largely reflected one common method factor—mitigate but do not eliminate it.

Second, the three-wave design supports tests of indirect associations but does not establish mediation in the causal sense. Temporal precedence of predictors over outcomes is necessary but not sufficient for causal mediation; without baseline (T1) measures of the mediators and outcomes we cannot rule out reverse or reciprocal influence or stable third variables, and we cannot distinguish prospective change from pre-existing individual differences. The bootstrapped “indirect effects” should accordingly be read as indirect *associations* consistent with—but not confirmatory of—the hypothesized pathways. Causal inference requires baseline-controlled designs (e.g., RI-CLPM) and experimental manipulation of offloading mode, which we did not undertake.

Third, the core mechanisms are incompletely measured. We invoke self-determination theory to motivate the motivational pathway but did not measure satisfaction of the autonomy and competence needs that the theory posits as the operative mechanism; intrinsic motivation is their proximal consequence, not the need-satisfaction process itself. Likewise, cognitive agency transfer is theorized as a gradual process but was measured as a single-occasion state appraisal, indexing how far the process has progressed rather than its dynamics. Future designs should include need-satisfaction measures and repeated within-person assessment of agency transfer.

Fourth, although we delineate the new constructs conceptually from adjacent ones (Section 2.2; Appendix A), we did not establish *empirical* discriminant validity against measured instruments. The new scales should be tested against established measures of AI dependency (Yu et al., 2024), automation complacency, blind trust, critical AI literacy, and self-regulated learning before strong claims of construct novelty are warranted.

Fifth, our sample comprised primarily Chinese university students and early-career workers (*M*_age_ = 23.69), and the worker subsample was small (*n* = 73). Although subgroup analyses (Section 4.7) showed the same overall pattern across students and workers, the one reliable group difference—a stronger motivational cost among workers—signals that task context, expertise, and AI-use demands may matter and deserve adequately powered, context-specific study. Participants also reported on AI use across heterogeneous tasks rather than a single standardized task; this heterogeneity increases ecological validity but leaves open which kinds of cognitive offloading drive the observed associations. Replication across age groups, occupations, cultures, and controlled task contexts is needed.

Finally, we note that we did not directly test whether users can or cannot *detect* maladaptive AI use; the dissociation between immediate benefit and downstream correlates (Section 5.1.3) is consistent with, but does not demonstrate, such a detection failure.

### 5.5 Conclusion

This study provides three-wave time-lagged correlational evidence (*N* = 589) that the manner of cognitive offloading to generative AI—dependent vs. autonomous—is differentially associated with *perceived* downstream cognitive functioning through cognitive agency transfer and intrinsic motivation, a pattern that proved robust to a latent-variable specification and across student and early-career-worker subsamples. Both modes were associated with comparable short-term benefits; whether this leaves users unable to detect potentially maladaptive patterns is a question our design raises but does not test. These findings highlight the manner of AI engagement, rather than its frequency, as a factor warranting attention—while underscoring that the evidence concerns subjective appraisals, not demonstrated cognitive ability, and associations, not causal effects. Baseline-controlled and experimental designs with performance-based outcomes are needed to move from this correlational foundation toward causal and ability-level claims.

## Data Availability

The datasets analyzed for this study are not publicly available because they contain participant-level survey responses. De-identified data and analysis materials are available from the corresponding author upon reasonable request, subject to institutional ethics requirements.
