# Supplementary material for: Not all cognitive offloading is equal: distinguishing dependent and autonomous offloading to generative AI
Source: Front Psychol. 2026 Jul 16;17:1878629. doi: 10.3389/fpsyg.2026.1878629 (PMC13420724; doi:10.3389/fpsyg.2026.1878629)
Supplement: Supplementary file 1 [file Supplementary_file_1.pdf]

Supplementary Material

Not All Cognitive Offloading Is Equal: Distinguishing Dependent and Autonomous Offloading to Generative AI

Supplementary Appendix A. Construct Boundary Summary

Table 1 summarizes how the core constructs in this study relate to, and differ from, adjacent constructs in the literature.

Table 1: Construct Definitions and Boundaries with Adjacent Constructs

Construct	Definition in this study	Adjacent construct	Key difference
Dependent cognitive offloading	Delegating core thinking to AI; accepting AI outputs with minimal evaluation	AI dependency (Yu et al., 2024)	AI dependency is a person-level, addiction-like reliance indexed by usage volume and loss of control; dependent offloading is task-level and frequency-agnostic—it describes *how* thinking is allocated within an episode, not how much AI is used overall
Dependent cognitive offloading	(as above)	Automation complacency; blind trust / over-trust (Parasuraman & Manzey, 2010)	Complacency and over-trust are attitudes/attentional states directed *at the tool* (reduced monitoring; miscalibrated confidence); dependent offloading is an allocation of the user's *own* cognitive operations
Autonomous cognitive offloading	Using AI as a scaffold while retaining evaluative authority and cognitive ownership	Critical AI literacy; self-regulated learning (SRL)	Critical AI literacy is a stable knowledge/skill competence and SRL a broad goal–monitor–reflect cycle over whole episodes; autonomous offloading is the narrower behavioral enactment within a single AI-assisted act that literacy/SRL may enable but do not guarantee
Cognitive agency transfer	Progressive ceding of cognitive governance—decisions about relevance, evaluation, and conclusions—to AI	Automation trust; blind trust; reduced self-efficacy	Trust concerns confidence in the tool's accuracy; agency transfer concerns *who steers the thinking process*. One can trust outputs yet retain governance, or distrust them yet defer governance—the two are separable

Note. These boundaries are conceptual. Empirical discriminant validity against adjacent constructs remains to be established in future measurement work.

Supplementary Appendix B. Scale Items

Note. All items were rated on a 5-point Likert scale (1 = strongly disagree; 5 = strongly agree). Items were administered in Chinese; English translations are provided below.

Dependent Cognitive Offloading (T1)

1. When using AI for a task, I prefer to let AI generate the complete answer directly.
2. I tend to accept AI-generated content without much modification.
3. I rely on AI to organize my ideas and structure my work.
4. When AI provides a solution, I usually adopt it as my final answer.

Autonomous Cognitive Offloading (T1)

1. When using AI for a task, I use AI suggestions as a starting point for my own thinking.
2. I compare AI-generated content with my own ideas before deciding what to use.
3. I use AI to explore different perspectives, but I form my own conclusions.
4. AI helps me think more broadly, but I make the final decisions myself.

Metacognitive Monitoring (T1)

1. While using AI, I regularly check whether I truly understand the content rather than just accepting it.
2. I am aware of when I am relying too heavily on AI instead of thinking for myself.
3. I monitor whether my use of AI is helping me learn or just helping me finish tasks.
4. I reflect on whether I could explain or reproduce AI-generated content on my own.

Task Complexity (T1)

1. The tasks for which I use AI often require integrating multiple perspectives.
2. The tasks I use AI for tend to be open-ended with no single correct answer.
3. My AI-assisted tasks often require weighing competing considerations.
4. The tasks for which I use AI usually involve complex information.

Cognitive Agency Transfer (T2)

1. I increasingly rely on AI to determine which information is most important for my tasks.
2. I tend to let AI decide how to approach a problem rather than planning it myself.
3. I defer to AI's judgment about which perspectives or arguments are strongest.
4. I find myself trusting AI's conclusions more than my own initial analysis.

Intrinsic Motivation (T2)

1. I feel genuinely interested in thinking through the problems I work on, even when AI could do it for me.
2. I enjoy the process of figuring things out on my own in my work domain.
3. I find it satisfying to deeply understand the material I work with.
4. I am curious to explore ideas beyond what AI suggests to me.

Immediate Benefit (T2)

1. Using AI allows me to complete tasks faster and with less effort.
2. AI helps me produce higher quality work in the short term.
3. I feel more efficient when I use AI for cognitive tasks.
4. AI reduces my mental burden when working on complex tasks.

Subsequent Autonomous Capability (T3)

1. I feel confident that I can handle similar tasks on my own without AI.
2. I believe I have developed the skills to work independently in this domain.
3. I can effectively complete tasks in this area even without AI assistance.
4. I feel capable of producing good work without relying on AI.

Creativity (T3)

1. I can generate novel ideas and approaches when working on tasks in this domain.
2. I am able to come up with original solutions to problems in my work area.
3. I can think of creative alternatives that go beyond conventional approaches.
4. I produce work that others would consider innovative or imaginative.

Deep Processing (T3)

1. I carefully evaluate and think deeply about information rather than accepting it at face value.
2. I try to understand the underlying logic and reasoning behind the information I encounter.
3. I actively integrate new information with what I already know.
4. I critically examine arguments and evidence before forming conclusions.

Independent Judgment (T3)

1. I can independently evaluate information and reach well-reasoned conclusions.
2. I feel confident forming my own opinions even when they differ from popular views.
3. I can assess the quality of different arguments without needing external guidance.
4. I trust my own analytical abilities when making important judgments.
